# Alpha-synuclein biology in Lewy body diseases

**DOI:** 10.1186/s13195-014-0073-2

**Published:** 2014-10-27

**Authors:** Woojin Scott Kim, Katarina Kågedal, Glenda M Halliday

**Affiliations:** 1 Neuroscience Research Australia, Barker Street, Randwick 2031, NSW, Australia; 2 School of Medical Sciences, University of New South Wales, Sydney 2052, NSW, Australia; 3 Department of Clinical and Experimental Medicine, Linköping University, Linköping, SE-581 85, Sweden

## Abstract

α-Synuclein is an abundantly expressed neuronal protein that is at the center of focus in understanding a group of neurodegenerative disorders called α-synucleinopathies, which are characterized by the presence of aggregated α-synuclein intracellularly. Primary α-synucleinopathies include Parkinson’s disease (PD), dementia with Lewy bodies and multiple system atrophy, with α-synuclein also found secondarily in a number of other diseases, including Alzheimer’s disease. Understanding how α-synuclein aggregates form in these different disorders is important for the understanding of its pathogenesis in Lewy body diseases. PD is the most prevalent of the α-synucleinopathies and much of the initial research on α-synuclein Lewy body pathology was based on PD but is also relevant to Lewy bodies in other diseases (dementia with Lewy bodies and Alzheimer’s disease). Polymorphism and mutation studies of *SNCA*, the gene that encodes α-synuclein, provide much evidence for a causal link between α-synuclein and PD. Among the primary α-synucleinopathies, multiple system atrophy is unique in that α-synuclein deposition occurs in oligodendrocytes rather than neurons. It is unclear whether α-synuclein originates from oligodendrocytes or whether it is transmitted somehow from neurons. α-Synuclein exists as a natively unfolded monomer in the cytosol, but in the presence of lipid membranes it is thought to undergo a conformational change to a folded α-helical secondary structure that is prone to forming dimers and oligomers. Posttranslational modification of α-synuclein, such as phosphorylation, ubiquitination and nitration, has been widely implicated in α-synuclein aggregation process and neurotoxicity. Recent studies using animal and cell models, as well as autopsy studies of patients with neuron transplants, provided compelling evidence for prion-like propagation of α-synuclein. This observation has implications for therapeutic strategies, and much recent effort is focused on developing antibodies that target extracellular α-synuclein.

## 1 Introduction

α-Synuclein is a 140 amino acid, natively unfolded protein predominantly localized in the presynaptic terminals of neurons. In the past two decades α-synuclein has been the center of focus in understanding the etiology of a group of overlapping neurodegenerative disorders called α-synucleinopathies, which includes Parkinson’s disease (PD), Parkinson’s disease dementia (PDD), dementia with Lewy bodies (DLB), multiple system atrophy (MSA) and a number of less-well characterized neuroaxonal dystrophies. α-Synuclein is encoded by the *SNCA* gene on 4q21, and was first identified as the nonamyloid component of β-amyloid plaques in the brain of patients with Alzheimer’s disease (AD) [1]. Although AD is pathologically quite distinct from α-synucleinopathies, α-synuclein aggregates have been found in the majority of AD brains, mostly restricted to the amygdala [2],[3]. Despite much research into α-synuclein biology, the exact function of α-synuclein is still elusive. α-Synuclein is thought to play a role in maintaining a supply of synaptic vesicles in presynaptic terminals. The protein has also been suggested to be involved in regulating the release of the neurotransmitter dopamine in controlling voluntary and involuntary movements.

The universal feature of α-synucleinopathies is the presence of proteinaceous intracellular entities or bodies containing aggregates of α-synuclein. These bodies differ somewhat in appearance in different α-synucleinopathies, and are called Lewy bodies in PD and DLB [4], glial cytoplasmic inclusions in MSA [5] and axonal spheroids in neuroaxonal dystrophies [6]. Much evidence indicates that the mechanism underpinning α-synucleinopathies is the misfolding of α-synuclein into aggregates [4]. *In vitro* studies have shown that α-synuclein aggregates (that is, oligomers) cause a series of secondary processes leading to neuroinflammation, neurodegeneration and cell death [7]. Apart from the pathogenic dogma of neurotoxicity of aggregated α-synuclein, loss of α-synuclein monomers (that is, loss of function) from their physiological location may also contribute to neurodegeneration [8]. A radical idea of prion-like propagation has been proposed for α-synuclein transmission between cells. New developments in α-synuclein transmission highlight the importance of extracellular α-synuclein in therapeutic strategies. In this review we will discuss α-synuclein biology, α-synucleinopathies and recent developments in α-synuclein disease mechanisms and therapies.

## 2 α-Synuclein biology

α-Synuclein is abundantly expressed in the human brain, making up as much as 1% of protein content in the cytosol. This protein is expressed throughout the brain, with high levels in the neocortex, hippocampus, substantia nigra, thalamus and cerebellum. It is predominantly expressed in neurons and to a lesser extent in glial cells. Apart from the predominant 140 amino acid protein, there are at least two other alternatively spliced variants of the protein; the 126 amino acid and 112 amino acid variants that lack exon 3 and exon 5, respectively [9]. The α-synuclein protein has three distinct structural domains. The amphipathic N-terminal region (residues 1 to 60) contains 11 amino acid repeats including the consensus sequence KTKEGV, which is important in α-helix formation [10]. The central hydrophobic region (residues 61 to 95) contains the nonamyloid component region, which is important in protein aggregation [4]. Finally, the C-terminal region (residues 96 to 140) is highly acidic and proline rich.

α-Synuclein is encoded by the *SNCA* gene. PD genome-wide association studies have shown that single nucleotide polymorphisms in *SNCA* are strongly associated with an increased risk for idiopathic PD [11]-[14]. The *SNCA* missense mutation Ala53Thr was the first causal mutation identified in dominantly inherited PD [15]. Several *SNCA* missense mutations (for example, Glu46Lys, His50Gln, Gly51Asp and Ala30Pro) have since been identified in dominantly inherited PD [16]-[19]. In 1998 Conway and colleagues demonstrated that *SNCA* missense mutations accelerated α-synuclein fibril formation *in vitro*, implicating α-synuclein misfolding and aggregation in PD pathogenesis [20]. *SNCA* duplication and triplication have also been identified in PD subjects [21]-[25].

Although the exact function of α-synuclein is unknown, α-synuclein is thought to play a role in maintaining a supply of synaptic vesicles in mature presynaptic terminals, because its expression was detected only after synaptic development [26]. *In vitro* knockdown studies showed that α-synuclein regulates the quantity of different pools of synaptic vesicles in mature neurons [26], influencing synaptic activity as a molecular chaperone in the formation of SNARE complexes [27], a requirement for presynaptic nerve terminal release of neurotransmitters [28]. In this way, α-synuclein may regulate the release of dopamine in controlling voluntary and involuntary movements, or might influence memory and cognitive function as shown in *SNCA* knockout mice [29]. This function of α-synuclein becomes more important during increased synaptic activity and aging, and could be a contributory factor in neurodegeneration.

## 3 Posttranslational modification of α-synuclein

Posttranslational modification of α-synuclein is prevalent and altered α-synuclein proteins impact on a number of pathological processes, including α-synuclein aggregation, Lewy body formation and neurotoxicity. The most common posttranslational modification of α-synuclein is phosphorylation, which occurs predominantly at serine residues S129 and, to a lesser extent, S87 and at tyrosine residues Y125, Y133 and Y135 [30],[31]. In DLB brains, approximately 90% of insoluble α-synuclein is phosphorylated at S129 compared with only 4% in soluble cytosolic α-synuclein [32], implicating phosphorylated α-synuclein in the process of α-synuclein aggregation.

The second most common posttranslational modification of α-synuclein is ubiquitination – the attachment of ubiquitin to α-synuclein at lysine residues. Although α-synuclein contains 15 lysine residues, α-synuclein isolated from Lewy bodies shows that the protein is ubiquitinated mainly at K6, K10 and K12 residues. Ubiquitination of α-synuclein causes changes in α-synuclein function/activity, impacting on α-synuclein localization and α-synuclein degradation processes [33]-[35].

Another common posttranslational modification of α-synuclein is nitration – the attachment of a nitro molecule to α-synuclein at tyrosine residues (Y39, Y125, Y133 and Y136). High concentrations of nitrated α-synuclein are found in Lewy bodies [36]. Nitration of α-synuclein is enhanced under conditions of elevated oxidative stress, which is widely regarded as an important factor in Lewy body diseases. *In vitro* studies have shown that nitration of α-synuclein induced α-synuclein oligomer formation and mitochondrial impairment, leading to apoptosis via the integrin pathway [37]. In a PD cell model, nitration of α-synuclein (via increased nitric oxide production) caused increases in the level of neurotoxic α-synuclein species and cell death [38].

## 4 Prion-like propagation of α-synuclein

In 2008, two autopsy studies of patients with PD who survived more than 10 years after receiving successful transplants of embryonic dopamine neurons to treat their disease observed that the surviving transplanted neurons had α-synuclein accumulation in typical Lewy bodies [39],[40]. The only way these neurons could have such pathology was by a propagating mechanism, a concept of transmission more commonly associated with prion diseases [41]. It should be noted that Braak and colleagues had in 2003 proposed a transmissible mechanism for α-synuclein propagation based on observations that the disease seemed to start in the nose and/or gut and progress to invade the brain in a staged manner [42],[43]. A number of subsequent studies in animal and cell culture models have proven this concept of transmission of α-synuclein between neurons, showing that exogenous α-synuclein induces Lewy body pathology along neuroanatomical pathways in the brain (for example [44]-[48]). It should be noted that it is the conformation of the protein that is transmitted to endogenous protein residing within neurons, as in mouse models the aggregates from exogenous sources disappear in about a week with endogenous aggregates beginning around 3 months later [49]. This observation suggests that a particular strain of α-synuclein is transmitted between neurons.

Consistent with the concept of different prion strains [50], a number of studies have now identified and characterized different strains of α-synuclein. Strains made *in vivo* exhibit fundamentally different properties, including the packing of their building blocks and growth and amplification properties, as well as their tropism, cellular binding and penetration properties and toxicity [51],[52]. These differences can be exaggerated by modifying the solution concentration, molecular crowding, agitation, temperature, pH and ionic strength [53]. Exogenous factors that accelerate the *in vitro* aggregation of α-synuclein include agrochemicals, polycations, histones, metal ions, glycosaminoglycans, sodium dodecyl sulfate and organic solvents, while factors that inhibit α-synuclein aggregation include small chemical compounds, heat shock proteins, PAMAM dendrimers, β-synuclein and γ-synuclein, catecholamines, phospholipids, rifampicin, trehalose and oxidative modifications [53]. The combination of different factors may impact on the strains of α-synuclein in different people and may explain some of the heterogeneity that is known both clinically and pathologically, and especially in the dynamics of the different types of Lewy body diseases [54]. Morphological and structural differences have been noted in patients with Lewy bodies consistent with the concept of different α-synuclein strains – Lewy bodies in the brainstem are morphologically different from those in the cortex [55], and conformationally different strains of α-synuclein have been identified from cortical tissue samples of patients with PD depending on the presence or absence of Alzheimer pathologies [52].

## 5 Binding and interaction of α-synuclein with lipid membranes

Under normal conditions, α-synuclein exists as a randomly structured and natively unfolded protein and remains as a monomer within the cytoplasm. Under pathological conditions, however, α-synuclein undergoes structural/conformational changes causing the monomers to aggregate with each other and become insoluble. Much evidence suggests that changes to the α-synuclein structure and properties are initiated when the protein binds and interacts with lipid surfaces, such as lipid droplets, phospholipid bilayers or lipid membranes. When α-synuclein monomers, isolated from human neurons, were exposed to synthetic lipid membranes, they readily bound to the membrane surface and formed dimers and oligomers [56],[57]. Such an interaction is thought to induce a dramatic change in α-synuclein structure from its unfolded form to a folded α-helical secondary structure [57]. The imperfect repeats of 11 amino acids present in α-synuclein, similar to the amphipathic α-helical motif common to apolipoproteins and other lipid-binding proteins, appear to play an important role in the lipid membrane binding process [58]. What is significant about such a change is that the α-helical form of α-synuclein is prone to forming different types of oligomers, the species that are thought to be toxic to cells. The lipid composition of membranes has been shown to affect the binding/interaction of α-synuclein to the membrane and subsequent oligomerization [56],[59]. α-Synuclein is thought to preferentially bind to regions of membranes that are enriched in lipids [60]. These regions are called lipid rafts and are characterized by high concentrations of cholesterol and sphingolipids and altered surface charge that may favor α-synuclein binding. The lipid rafts appear to serve as a platform that promotes α-synuclein binding and oligomerization.

Contrary to overwhelming evidence that α-synuclein exists as an unfolded monomer in the cytosol, Bartels and colleagues reported that endogenous α-synuclein exists predominantly as a folded tetramer (~58 kDa) [61]. The explanation provided by the authors for this apparent difference is that most studies claiming the unfolded monomer hypothesis commonly use sample heating and denaturing gels to analyze α-synuclein, whereas the authors used nondenaturing conditions. They have also provided evidence by other means – that is, scanning transmission electron microscopy and cell cross-linking – to confirm the prevalence of α-synuclein tetramer in neurons and human brain tissues [61]. Bartels and colleagues proposed that since α-synuclein tetramers are less likely to form aggregates, the tetramers first undergo destabilization prior to forming aggregates. The authors suggested that stabilizing the physiological tetramers could reduce α-synuclein pathogenicity in PD and other α-synucleinopathies.

## 6 Dementia with Lewy bodies

DLB was initially identified as a dementia syndrome with Lewy body pathology [62], which is now incorporated in the *Diagnostic and Statistical Manual* criteria as a clinical disease entity (neurocognitive disorder with Lewy bodies). Current objective data suggest that the sensitivity of accurate clinical diagnosis is very low, however, with most clinical cases identified actually having AD rather than DLB at autopsy [63]-[68], and therefore current diagnostic criteria for DLB exclude cases with coexisting AD pathology [62]. Although DLB remains easy to identify pathologically with different cellular pathologies differentiating it from other dementia syndromes, pathological identification using only Lewy body pathology has been shown to be inaccurate due to overlap with patients without dementia symptoms. Current neuropathological criteria state that neurocognitive syndromes with Lewy bodies are most likely when Lewy bodies are prevalent in at least limbic brain regions, but are also often found in association neocortices [69]. A number of studies have shown that a combination of cellular pathologies, which include α-synuclein and β-amyloid deposition as well as dopamine denervation, assist with differentiating this dementia syndrome from others [54]. Approximately 25% of DLB patients display significant parkinsonian symptoms at the onset of disease, consistent with an early dopamine denervation, whereas 25% of DLB patients never develop any parkinsonian symptoms and have less significant dopamine loss. DLB is best conceptualized as a dominant dementia syndrome with multiple pathologies that include Lewy bodies and more frequently has multiple pathologies compared with AD [70]. The diversity of clinical phenotypes associated with DLB is likely to reflect the timing and different combinations of these pathologies within different brain regions.

Because of the difficulty in obtaining clinically proven cases with pathological DLB, studies of the underlying molecular changes in the brain are rare. Interesting pathological differences have been noted – the longer the duration of parkinsonism prior to dementia onset, the less severe the cortical α-synuclein and β-amyloid deposition as well as the cortical cholinergic deficit [71]. DLB patients present significant cholinergic deficits [72]-[74] and a decrease in serum α-synuclein [75].

## 7 Parkinson’s disease and Parkinson’s disease dementia

In contrast to DLB, which is a dominant dementia syndrome, PD is a dominant movement disorder characterized by the presence of two of four cardinal signs (that is, bradykinesia, rigidity, resting tremor, gait instability) that are responsive to levodopa therapy [76]. Current neuropathological criteria require moderate to severe loss of pigmented dopamine neurons in the substantia nigra along with Lewy bodies at least in the brainstem [69]. PDD was defined in 2007 as a dementia syndrome in patients with an initial diagnosis of PD for more than 1 year [77] and, as stated above for DLB, the cognitive symptoms are thought to occur when Lewy bodies are prevalent in at least limbic brain regions, but often also in association neocortices [69]. A smaller proportion of people with PDD have multiple pathologies [78] as observed in most DLB cases (see above).

Changes in the phosphorylation and solubility of α-synuclein occur prior to Lewy body formation in PD and PDD [79]-[81]. In terms of solubility, the amount of soluble α-synuclein is not substantially increased and actually decreases slightly over the course of PD [79],[82]. The levels of phosphorylation of α-synuclein greatly increase prior to Lewy body formation [79]-[81] and the Lewy body formation correlates with an enhanced lipid association of α-synuclein [79]. In a longitudinal study of patients with PD it took an average 13 years for the propagation of Lewy body aggregates to reach limbic brain regions, and 18 years before aggregates occurred in association cortices in 50% of PD cases [83]. These studies show that the intracellular changes in α-synuclein take considerable time to propagate and that posttranslational modifications of α-synuclein are substantial prior to its irreversible fibrilization.

## 8 Multiple system atrophy

MSA is a rapidly progressive neurodegenerative disease characterized by the clinical triad of parkinsonism (similar to PD), cerebellar ataxia and autonomic failure. The distribution of pathology classically encompasses three functional systems in the central nervous system – the striatonigral system, the olivopontocerebellar system and the autonomic system – impacting on movement, muscle control, blood pressure, heart rate and bladder function [84],[85]. Like PD and DLB, the dominant histopathology of MSA is the presence of misfolded and fibrillar α-synuclein in the cytoplasm. However, unlike PD and DLB, the principal site for α-synuclein deposition is in the oligodendrocytes rather than neurons. Based on current information, the sequence of pathological events in MSA is now recognized as myelin dysregulation first, followed by demyelination and then neurodegeneration and loss of neurons [86]-[88]; neurodegeneration therefore appears to be a secondary effect in MSA.

No causal mutations or multiplications of the coding sequence of α-synuclein have been identified in MSA cases [89]-[91], although the search is not exhaustive because MSA is a rare disease. Earlier studies, based on small numbers of MSA cases, have reported that genetic variants of *SNCA* were associated with MSA [92]-[94]; however, a recent pioneering genome-wide association study of 918 MSA cases and 3,884 controls found no risk-conferring loci on the *SNCA* gene [95]. Posttranslational modification studies of α-synuclein in MSA have shown that phosphorylation and ubiquitination are implicated in the deposition of α-synuclein [96], although no definitively causative relationships have yet been established. Furthermore, the origin of α-synuclein in oligodendrocytes remains stubbornly enigmatic. Although the evidence of significant physiological expression of α-synuclein in mature oligodendrocytes is conflicting [97]-[99], it has been proposed that upregulation of the *SNCA* gene in these cells could be the cause of α-synuclein aggregation. Nevertheless, successful animal models of MSA, which recapitulate both neuropathological and clinical features, have been generated by overexpression of α-synuclein in the oligodendrocytes [96],[100],[101]. Alternatively, aberrant uptake of α-synuclein from the extracellular environment has also been proposed as a possible mechanism of α-synuclein aggregation in oligodendrocytes [97],[102],[103].

## 9 Lewy body pathology in Alzheimer’s disease

Although Lewy bodies are the pathological hallmark of PD and DLB, recent studies suggest a considerable proportion of AD brains show α-synuclein pathology. In a recent study of 22 clinically diagnosed cases of AD, 10 were found to have α-synuclein immunoreactive Lewy bodies by subsequent pathological examination [104]. Other studies showed that as many as one-half of patients with AD, including both sporadic and familial cases, have α-synuclein aggregates [2],[105]-[107]. In these studies, α-synuclein aggregates were mostly restricted to the amygdala, implying that the spread of α-synuclein inclusions is different to that of PD. Lewy pathology in AD has also been reported to be formed mainly in the cell body of neurons, and not in the axonal terminals and dendrites as in PD [107],[108]. The Lewy pathology therefore possibly mirrors a nonspecific end stage of AD. However, genetic or lifestyle factors might prime neurons to accumulate α-synuclein aggregates in a subset of AD patients, and thus α-synuclein aggregates might reflect a causal pathogenic mechanism in AD.

Several studies show that high levels of AD pathology are often observed in patients with PD and DLB [78] and correlate with the decline in cognitive function more than the amount of α-synuclein aggregates [109]-[111]. Interestingly, PD/DLB cases with AD pathology have higher α-synuclein levels in cortical and limbic areas than cases without AD pathology [112], which implies a possible interaction between α-synuclein and AD pathology in these disorders. How the pathologies of α-synuclein, β-amyloid and tau relate to each other in PD and AD is poorly understood. Recent work using a transgenic mouse model of DLB-AD provides some clues to the interaction between β-amyloid, tau and α-synuclein [113]. This mouse model was generated from a cross between 3 × Tg-AD mice and mice that express the A53T mutation in α-synuclein [114]. The DLB-AD mice exhibited accelerated cognitive decline, compared with 3 × Tg-AD mice alone, with more severe β-amyloid, tau and α-synuclein pathologies [113]. These data suggest that the three pathologies interact and somehow enhance each other, resulting in accelerated cognitive dysfunction.

## 10 Therapeutic strategies

Because of the marked cholinergic deficit associated with DLB (see above), cholinesterase inhibitors are routinely used for clinical improvement [115]. In PDD these agents have been shown to improve cognitive function, behavioral disturbances and activities of daily living [115]. Their effect in DLB is less clear [115], potentially because DLB is poorly diagnosed clinically and often has multiple underlying pathologies (see above). Interestingly, successful treatment with cholinesterase inhibitors was shown to decrease β-amyloid deposition in a small study of DLB patients [116], suggesting that these drugs have mechanistic as well as symptomatic effects. Considering the molecular events surrounding α-synuclein deposition, a number of strategies are being developed [117],[118]. These strategies include small anti-aggregating molecules and chaperones [119]-[123], but perhaps the most promising strategy is the development of antibody therapies for α-synuclein. These therapies target extracellular α-synuclein binding the protein to reduce its self-aggregation and increase its clearance, with a number of antibodies already in production [124]-[127]. Another promising development is the use of the β-lactum antibiotic ceftriaxone as a therapeutic agent to block α-synuclein aggregation [128], although the macrocyclic antibiotic rifampicin has not been successful in MSA [129].

## 11 Conclusions

The assessment of different α-synucleinopathies focuses on a variety of mechanisms that affect the pathogenesis of Lewy body diseases. While all α-synucleinopathies are characterized by α-synuclein aggregates with similar posttranslational modifications and lipid associations, the cell type involved, their location and their association with other protein depositions vary substantially, and recent data suggest that perhaps the strain of α-synuclein involved may also differ. An increase in α-synuclein is hypothesized to precipitate the protein’s aggregation, and this is evident in some familial forms of PD, but the precipitating events for most of the α-synucleinopathies remain to be determined. It is clear for Lewy body disorders that the neuronal propagation can be slow or rapid, and is impacted on by AD pathology; however, Lewy bodies in AD are focused in the amygdala, suggesting that the initiating region of α-synuclein aggregation in the brain can be diverse. Importantly, the concept of propagation of α-synuclein pathology between neurons has resulted in the development of new therapies that target this mechanism with the potential to halt or slow this aspect of Lewy body diseases.

**Note**

This article is part of a series on *Lewy Body Dementia*, edited by Ian McKeith and James Galvin. Other articles in this series can be found at http://alzres.com/series/LewyBodyDementia.

## Abbreviations

AD: Alzheimer’s disease

DLB: Dementia with Lewy bodies

MSA: Multiple system atrophy

PD: Parkinson’s disease

PDD: Parkinson’s disease dementia

## Competing interests

The authors declare that they have no competing interests.
